# Basophil activation test in allergic patients to platins undergoing rapid desensitization

**DOI:** 10.1186/2045-7022-4-S3-P34

**Published:** 2014-07-18

**Authors:** Pedro Giavina-Bianchi, Joana Caiado, Matthieu Picard, Violeta Régnier Galvão, Mariana Castells

**Affiliations:** 1 Harvard Medical School, Division of Rheumatology, Allergy and Immunology, USA; 2 Harvard Medical School, Division of Rheumatology, Allergy and Immunology, Department of Medicine, BWH, USA; 3 University of Sao Paulo, Clinical Immunology and Allergy, Brazil

## Background

Desensitization (DST) has become a cornerstone in the management of immediate hypersensitivity reactions (HSRs) to chemotherapic agents. It is the only effective procedure for overcoming HSRs to first-line therapy, thus representing an important advance in patients' treatment and prognosis. Nevertheless, there are still no good biomarkers to monitor DST safety and effectiveness. The basophil activation test (BAT) has greater applicability in difficult cases with inconclusive diagnosis and when other diagnostic tests (skin tests and serum specific IgE) have not been standardized, as in drug-induced reactions. The main goal of our study was to assess BAT as a test to monitor rapid DST in patients allergic to platins.

## Method

We studied 7 oncologic patients who presented platin allergy and 4 healthy volunteers who had never been exposed to platins. We performed the BAT immediately before DST to platins, assessing CD 203c and CD63 expression on basophils. All patients were evaluated at least in 2 different DST.

## Results

BAT was positive in 6 patients (85.7%), with increased expression of CD203c and CD63 in 6 and 3 patients, respectively. Subsequent BAT analysis in different DST procedures showed that the test remained positive with an even greater expression of CD203c and CD63 on basophils after platins exposure. Some patients with positive BAT reacted during DST, in spite of being premedicated, showing the correlation between BAT results and clinical outcomes. Further investigation is necessary to determine BAT predictive values and its clinical applicability.

## Conclusion

We developed a BAT to platins that presented good sensitivity in our study. Short-term DST to platinum drugs does not induce persistent hyporresponsiveness on basophils, highlighting the need to maintain DST in allergic patients to platins.

